# Association Between Medicaid Expansion and Insurance Status, Risk Group, Receipt, and Refusal of Treatment Among Men with Prostate Cancer

**DOI:** 10.3390/cancers17030547

**Published:** 2025-02-06

**Authors:** Tej A. Patel, Bhav Jain, Edward Christopher Dee, Khushi Kohli, Sruthi Ranganathan, James Janopaul-Naylor, Brandon A. Mahal, Kosj Yamoah, Sean M. McBride, Paul L. Nguyen, Fumiko Chino, Vinayak Muralidhar, Miranda B. Lam, Neha Vapiwala

**Affiliations:** 1 Department of Healthcare Management & Policy, University of Pennsylvania, Philadelphia, PA 19104, USA; tapatel@wharton.upenn.edu; 2 Department of Health Policy, Stanford University School of Medicine, Stanford, CA 94305, USA; bhavjain@mit.edu; 3 Department of Radiation Oncology, Memorial Sloan Kettering Cancer Center, New York, NY 10065, USA; janopaj@mskcc.org (J.J.-N.); mcbrides@mskcc.org (S.M.M.); 4 Department of Biology, Harvard University, Cambridge, MA 02138, USA; kkohli@college.harvard.edu; 5 Department of Medicine, University of Cambridge, Cambridge CB2 3EN, UK; sr932@cam.ac.uk; 6 Department of Radiation Oncology, Sylvester Comprehensive Cancer Center, University of Miami Miller School of Medicine, Miami, FL 33136, USA; bmahal@med.miami.edu; 7 Department of Radiation Oncology, H. Lee Moffitt Cancer Center, Tampa, FL 33612, USA; kosj.yamoah@moffitt.org; 8 Department of Radiation Oncology, Dana-Farber Cancer Institute, Brigham and Women’s Hospital, Harvard Medical School, Boston, MA 02115, USA; paul_nguyen@dfci.harvard.edu (P.L.N.); miranda_lam@dfci.harvard.edu (M.B.L.); 9 Department of Radiation Oncology, University of Texas MD Anderson Cancer Center, Houston, TX 77030, USA; fchino@mdanderson.org; 10 Kaiser Permanente Northwest, Portland, OR 97227, USA; vinayak.x.muralidhar@kp.org; 11 Department of Radiation Oncology, University of Pennsylvania, Philadelphia, PA 19104, USA

**Keywords:** Medicaid expansion, insurance status, risk group, treatment refusal, racial disparities

## Abstract

**Background**: Although the Patient Protection and Affordable Care Act (ACA) has been associated with increased Medicaid coverage among prostate cancer patients, the association between Medicaid expansion with risk group at diagnosis, time to treatment initiation (TTI), and the refusal of locoregional treatment (LT) among patients requires further exploration. **Methods**: Using the National Cancer Database, we performed a retrospective cohort analysis of all patients aged 40 to 64 years diagnosed with localized prostate cancer from 2011 to 2016. Difference-in-difference (DID) analysis was used to compare changes in insurance status, risk group at diagnosis, TTI, and the refusal of LT among patients residing in Medicaid expansion versus non-expansion states. In a secondary analysis, we used DID to compare changes in the above outcomes among racial minorities versus White patients living in expansion states. **Results**: Of the 112,434 patients with prostate cancer in our analysis, 50,958 patients lived in Medicaid expansion states, and 61,476 patients lived in non-expansion states. In the adjusted analysis, we found that the proportion of uninsured patients (adjusted DID: −0.87%; 95% confidence interval [95% CI]: −1.28 to −0.46) and patients who refused radiation therapy (adjusted DID: −0.71%; 95% CI: −0.95 to −0.47) decreased more in expansion states compared to non-expansion states. Similarly, we observed that the racial disparity of select outcomes in expansion states narrowed, as racial minorities experienced larger absolute decreases in uninsured status and the refusal of radiation therapy (RT) regimens than White patients following ACA implementation (*p* < 0.01 for all). However, residence in a Medicaid expansion state was not associated with changes in risk group at diagnosis, TTI, nor the refusal of LT (*p* > 0.01 for all); racial disparities in TTI were also exacerbated in expansion states following ACA implementation. **Conclusions**: The association between Medicaid expansion and prostate cancer outcomes and disparities remains unclear. While ACA implementation was associated with increased insurance coverage and decreased refusal of RT, there was no significant association with earlier risk group at diagnosis, TTI within 180 days, or refusal of LT. Similarly, racial minorities in expansion states had larger decreases in uninsured status and the refusal of RT regimens, as well as smaller increases in intermediate-/high-risk disease at presentation than White patients following ACA implementation, but experienced no significant changes in TTI. More research is needed to understand how Medicaid expansion affects cancer outcomes and whether these effects are borne equitably among different populations.

## Simple Summary

We sought to quantify the impact of Medicaid expansion on insurance status, stage at diagnosis, time to treatment initiation, and refusal of locoregional treatment among patients with prostate cancer, the second leading cause of cancer death among men in the United States. We found that while Medicaid expansion was associated with increased insurance coverage and decreased refusal of radiation therapy, there was no significant association with earlier risk group at diagnosis, treatment within 180 days, nor refusal of locoregional therapy. Similarly, racial minorities experienced no significant changes in time to treatment initiation following Affordable Care Act implementation compared to White patients. Ultimately, more research is needed to understand how Medicaid expansion affects cancer outcomes and whether these effects are borne equitably among different populations.

## 1. Introduction

The Patient Protection and Affordable Care Act (ACA) permitted states to expand Medicaid coverage to nonelderly adults with incomes at or below 138% of the federal poverty level [1]. Since the passage of the ACA, 41 states to date (including Washington, DC) have expanded Medicaid, and nearly 20 million individuals in the US have gained insurance coverage [2]. In addition to coverage, these expansions have been associated with increases in the affordability of treatment, more provision of preventative and screening services, as well as overall improved health outcomes [3,4,5,6,7,8]. However, despite these positive effects, prior studies have revealed that ACA implementation is associated with unintended consequences, such as lengthier wait times for treatment and the exacerbation of racial disparities in cancer stage at presentation [9,10,11]. Debate about the effects of Medicaid expansion is also ensuing at the state and federal levels, as the Supreme Court made expansion optional in 2012, and only 25 of the 41 states expanded Medicaid on the initial 1 January 2014 date [12]. This staggered pattern of expansion provides an opportunity to study the effects of policy adoption on disease-specific outcomes and determine if disparities exist.

The association of Medicaid expansion with the management of prostate cancer is particularly important given that it is commonly diagnosed, the second leading cause of cancer death among men in the US, and the fifth costliest cancer, accounting for almost USD 16 billion in estimated spending in 2020 [13,14]. Moreover, outcomes for patients with prostate cancer vary considerably by socioeconomic status and race/ethnicity [15]. For example, uninsured patients with prostate cancer are more likely to be non-white, present with metastatic disease, not receive definitive treatment for high-risk disease, and die from their cancer [16,17,18]. These effects are magnified in disadvantaged communities, as negative associations between a lack of insurance coverage and cancer-specific outcomes are larger in magnitude for low-income populations [19]. Additionally, Black patients are more often diagnosed with advanced disease at presentation and are also less likely to receive guideline-recommended treatment than White patients [20,21].

While research on the impact of ACA implementation has focused on changes in insurance status, less is known about the effects of Medicaid expansion on stage at diagnosis, time to treatment initiation (TTI), and, most notably, refusal of locoregional treatment. Furthermore, only a handful of studies have explored how the ACA has alleviated racial/ethnic disparities across the above outcomes, directly comparing how changes in these outcomes vary across sociodemographic strata. As a result, the objective of this study was to assess whether Medicaid expansion is associated with equitable changes in a range of prostate cancer outcomes.

## 2. Methods

### 2.1. Data Source and Study Population

The data for this study were provided by the National Cancer Database (NCDB), a dataset collected by the Commission on Cancer of the American College of Surgeons and the American Cancer Society that provides clinical oncology data sourced from over 1500 accredited facilities [22]. The NCDB is estimated to cover 58% of newly diagnosed prostate cancer cases and 72% of newly diagnosed malignancies in the US annually [23]. Our study population included men aged 40–64 years and diagnosed with low- (Gleason ≤ 6, prostate-specific antigen [PSA] < 10 ng/mL, and cT1-T2a), intermediate- (Gleason 7, PSA 10–20 ng/mL, or cT2b-T2c), or high-risk (Gleason 8–10, PSA  >  20 ng/mL, or cT3-T4) prostate cancer according to AUA/ASTRO guidelines from 2011 to 2016 [24,25]. The NCDB includes a variable that indicates whether a patient above 40 years old lives in an expansion or non-expansion state. Patients who were younger than 40 years were excluded from the analysis as expansion status for their state of residence was unavailable. Patients over 65 years old and those who were eligible for Medicare were also excluded.

**Figure 1:**
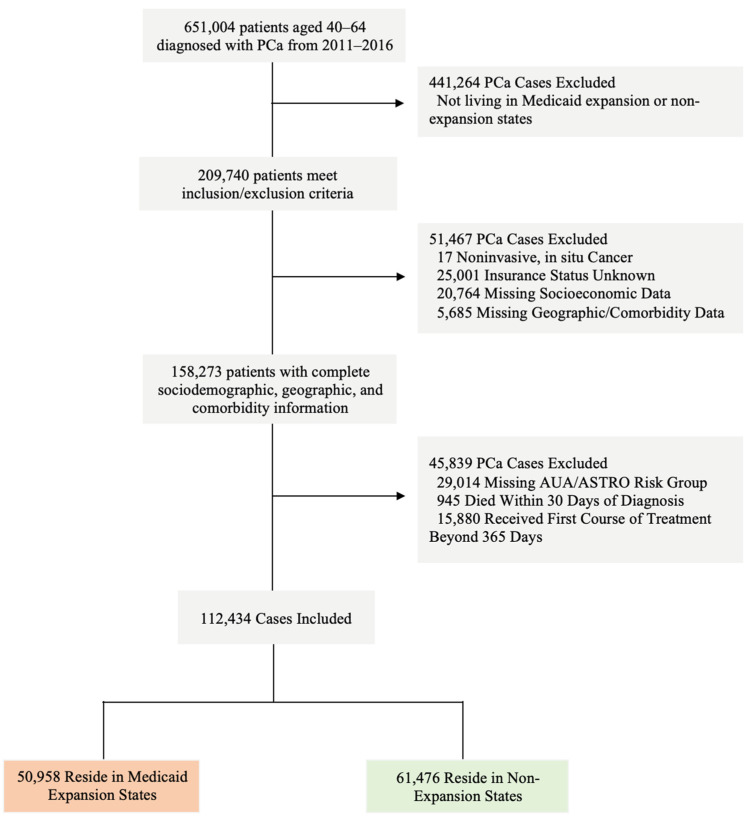
PRISMA diagram depicting cohort selection. Abbreviations: PCa: Prostate Cancer.

We identified 209,740 eligible patients with a new diagnosis of prostate cancer within the study period. We then excluded patients with noninvasive in situ cancers (<0.1%) and patients with missing sociodemographic, geographic, and comorbidity information (24.5%). For the analysis comparing AUA/ASTRO risk group at diagnosis as an outcome, those with unknown/missing risk group were excluded (13.8%). For the analysis of time to treatment initiation (TTI), patients who died within 30 days of diagnosis or received their first course of cancer-directed treatment beyond 365 days were excluded (8.0%). NCDB reports Medicaid expansion status based on the patient’s state of residence at the time of diagnosis as “non-expansion states”, “January 2014 expansion states”, “early expansion states (2010–2013),” and “late expansion states (after January 2014)” [26]. Our analyses only contain patients from the 19 states (KY, NV, CO, OR, NM, WV, AR, RI, AZ, MD, MA, ND, OH, IA, IL, VT, HI, NY, DE) that expanded Medicaid on January 1, 2014. We excluded patients from the six states that expanded Medicaid before (WA, CA, NJ, MN, DC, CT) and the seven states that expanded after (NH, IN, MI, PA, AK, MT, LA) January 2014, as these patients may dilute the initial effect of Medicaid expansion that took effect on 1 January 2014 (Figure 1, Table S1).

### 2.2. Study Design

In our primary analysis, we employed a quasi-experimental, difference-in-difference (DID) cross-sectional analysis comparing changes in insurance status, risk group at diagnosis, timely treatment, and treatment refusal among all prostate cancer patients residing in Medicaid expansion and non-expansion states before and after ACA implementation. This DID, cross-sectional analysis is typically used to estimate the specific effect of a policy intervention on select outcomes by comparing changes in those outcomes across a group that was exposed to the intervention versus a group that was not exposed to the intervention. DID has been used in a variety of studies examining the association of Medicaid expansion on health outcomes [10,11]. The exposure variable in our analysis was residence in a Medicaid expansion state, and the intervention was Medicaid expansion in January 2014.

In our secondary analysis, we used DID to compare changes in the above outcomes among White patients and racial minorities residing in Medicaid expansion states before and after ACA implementation. The exposure variable was race and ethnicity, and the intervention was Medicaid expansion in January 2014. The different racial and ethnic patient groups in our primary study cohort included Non-Hispanic White, Non-Hispanic Black, Hispanic, Asian American, and Native Hawaiian or Pacific Islander. Given small sample sizes, patients who identified as Chinese, Japanese, Filipino, Korean, Vietnamese, Laotian, Hmong, Kampuchean, Thai, and Asian Indian or Pakistani were collectively referred to as Asian American. Patients who identified as Native Hawaiian, Micronesian, Chamorran, Guamanian, Polynesian, Tahitian, Samoan, Tongan, Melanesian, Fiji Islander, New Guinean, and “Pacific Islander NOS” were grouped as Native Hawaiian or Pacific Islander.

Both primary and secondary analyses included patients diagnosed with low-, intermediate-, or high-risk prostate cancer from 1 January 2011 to 31 December 2016. Given that the aforementioned 19 states expanded their Medicaid programs on 1 January 2014, the pre-expansion period was defined as 1 January 2011 to 31 December 2013, and the post-expansion period was defined as 1 January 2014 to 31 December 2016.

### 2.3. Independent Variables

In the primary analysis, the main independent variables were residence in a Medicaid expansion state, diagnosis in the post-expansion period, and their respective interaction. In the secondary analysis, the main independent variables were race or ethnicity, diagnosis in the post-expansion period, and the interaction between the two. Only patients that resided in the “expansion” states were included in this secondary analysis.

### 2.4. Outcomes

In both analyses, the primary outcomes of interest included (1) insurance status (uninsured, Medicaid), (2) risk group at diagnosis (low-risk disease, intermediate-/high-risk disease), (3) time to first course of cancer-directed treatment (surgery, radiation, systemic, or other therapy within 30 days, 90 days, and 180 days of diagnosis), and (4) refusal of recommended locoregional treatment (surgery, radiation, or both surgery and radiation). In the secondary analysis, given that the primary independent variable was race or ethnicity, we sought to determine how changes in the above outcomes varied in reference to the racial population with the highest number of patients in the NCDB. If one racial group did not experience as great of a magnitude in change for a given outcome, then there is potential evidence of a persistent disparity in Medicaid expansion’s effect on patient outcomes.

### 2.5. Covariates

The relevant covariates that we adjusted for in the analysis included year of diagnosis, insurance status, age, Charlson–Deyo comorbidity coefficient score (CDCC) [27], zip-code-wide median household income, zip-code-wide education level, facility type (academic research program vs. community cancer program), distance from treatment facility, and rurality (urban vs. rural location). In the primary analysis with residence in a Medicaid expansion state as the exposure variable, each model was also adjusted for race and Hispanic ethnicity.

### 2.6. Statistical Analyses

Baseline clinical and sociodemographic characteristics were compared across patients residing in expanded and non-expanded states using Pearson’s χ^2^ test for categorical variables and the Mann–Whitney test for continuous variables. A multivariable linear regression was used to calculate adjusted DID estimates as a function of residing in an expansion state, diagnosis in the post-expansion period, and their respective interaction. For the secondary analysis, a multivariable linear regression calculated DID estimates as a function of race/ethnicity, diagnosis in the post-expansion period, and their interaction. The DID estimates represent the coefficient on the interaction term between expansion status (expansion vs. non-expansion) and time period (2011–2013 vs. 2014–2016) for the primary analysis or the coefficient on the interaction term between race/ethnicity and time period for the secondary analysis. Each regression was adjusted for relevant sociodemographic and geographic covariates. Linear models were used because they provide straightforward percentage point estimates for absolute change and have been used in previous DID studies [4,26]. All data were analyzed from June to September 2022 using Stata/SE, version 17.0 (StataCorp LLC, College Station, TX, USA). A two-sided *p* < 0.01 was considered statistically significant.

## 3. Results

### 3.1. Patient Characteristics

We identified 112,434 patients with complete sociodemographic, geographic, clinical, and treatment information diagnosed with prostate cancer from 2011 to 2016. We first compared baseline characteristics of patients that resided in Medicaid expansion (n = 50,958) and non-expansion (n = 61,476) states (Table 1). Compared with patients living in non-expansion states, patients residing in expansion states were less likely to be Black (15.9% vs. 23.8%) and more likely to be Hispanic (4.7% vs. 3.9%) and Asian American (1.5% vs. 0.4%). Additionally, those in expansion states were more likely to have Medicaid coverage (7.9% vs. 3.7%), live in metropolitan areas (87.8% vs. 81.7%), receive care at an academic center (62.2% vs. 45.9%), and reside closer, on average, to the treatment facility (11.7 mi vs. 14.1 mi).

### 3.2. Insurance Status

**Figure 2:**
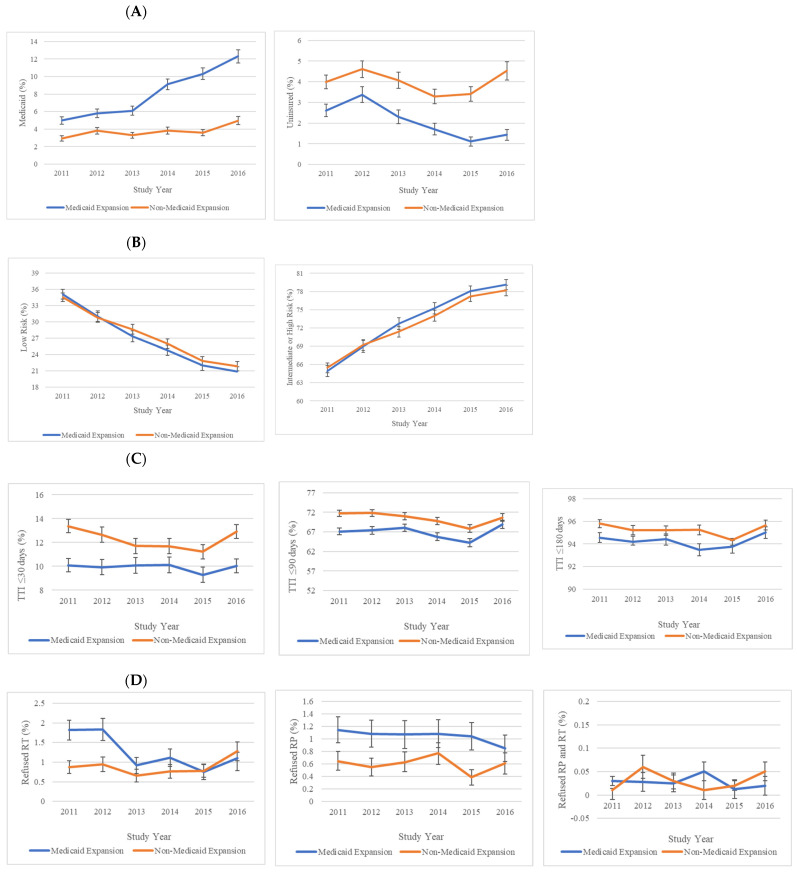
Adjusted trends in health insurance status (Panel (**A**)), risk group at initial diagnosis (Panel (**B**)), timely treatment (Panel (**C**)), and refusal of RP and/or RT (Panel (**D**)). Participants include patients aged 40–64 years old diagnosed with prostate cancer between 1 January 2011 and 31 December 2016 from the National Cancer Database. Error bars show 95% confidence intervals of estimated margins. A vertical red line denotes the enactment of the Affordable Care Act (ACA) and Medicaid Expansion. Abbreviations: Radiation Therapy (RT), Radical Prostatectomy (RP).

Unadjusted differences and adjusted DID estimates for changes in insurance status (Medicaid, uninsured) following Medicaid expansion are presented in Table 2. Parallel trends were observed for insurance status in the pre-expansion period (2011–2013, *p* > 0.01, Figure 2A). Both expansion states (difference: 4.93%; 95% CI: 4.47 to 5.40) and non-expansion states (difference: 0.77%; 95% CI: 0.47 to 1.07) had a significant increase in the proportion of Medicaid patients from the pre- to post-expansion period. The differences between groups were significant upon adjusting for sociodemographic and clinical factors (adjusted DID: 4.12%; 95% CI: 3.60 to 4.46). In the analysis where race/ethnicity was the exposure variable and ACA implementation was the intervention, only Non-Hispanic Black patients experienced greater improvements in Medicaid enrollment compared to White patients (adjusted DID: 2.72%; 95% CI: 2.08 to 3.35, Table 3) after expansion.

Moreover, both expansion (difference: −1.35%; 95% CI: −1.60 to −1.10) and non-expansion (difference: −0.51%; 95% CI: −0.82 to −0.20) states had significant decreases in the proportion of uninsured patients following Medicaid expansion. Upon adjustment, residence in an expansion state was associated with a percentage decrease in uninsured status following ACA implementation (adjusted DID: −0.87%; 95% CI: −1.28 to −0.46). Similarly, Non-Hispanic Black, Hispanic, and Asian American patients all experienced larger absolute decreases in uninsured status relative to White patients following Medicaid expansion (*p* < 0.01 for all, Table 3).

### 3.3. Risk Group at Diagnosis

Unadjusted differences and adjusted DID estimates for changes in risk group at diagnosis (low-risk, intermediate-/high-risk) following Medicaid expansion are presented in Table 2. Parallel trends were observed for risk group in the pre-expansion period (2011–2013, *p* > 0.01, Figure 2B). While the proportion of patients presenting with low-risk disease decreased for both expansion (difference: −15.2%; 95% CI: −16.0 to −14.5) and non-expansion (difference: −14.4%; 95% CI: −15.1 to −13.7) states, the adjusted difference between the two groups was not significant (adjusted DID: −0.62%; 95% CI: −1.60 to 0.36). Similarly, both expansion and non-expansion states experienced increases in the proportion of patients presenting with intermediate-/high-risk disease following the ACA, but the increases were not significant (adjusted DID: 0.62%; 95% CI: −0.36 to 1.60).

In the analyses where race/ethnicity interacted with diagnosis in the post-expansion period, we found that Non-Hispanic Black patients in expansion states experienced a smaller decrease in low-risk group disease at presentation (adjusted DID: 2.37%; 95% CI: 1.13 to 3.62) and a smaller increase in intermediate-/high-risk disease at presentation (adjusted DID: −2.37%; 85% CI: −3.62 to 1.13) than White patients after ACA implementation. Other racial and ethnic groups, including Hispanic, Asian American, and Native Hawaiian or Pacific Islander populations, had no relative differences in risk group at presentation following Medicaid expansion (*p* > 0.01 for all, Table 3).

### 3.4. Time to Treatment Initiation (TTI)

Unadjusted differences and adjusted DID estimates for changes in time-to-treatment initiation (TTI ≤ 30 days, ≤90 days, ≤180 days after diagnosis) following Medicaid expansion are presented in Table 2. Parallel trends were observed for TTI in the pre-expansion period (2011–2013, *p* > 0.01, Figure 2C). Expansion states experienced an increase in the proportion of patients with TTI ≤ 30 days (difference 0.69%; 95% CI: 0.16 to 1.22), while non-expanded states had a slight decrease (difference: −0.16%; 95% CI: −0.69 to 0.36). The adjusted difference between expansion and non-expansion states was not significant (adjusted DID: 0.63%; 95% CI: −0.12 to 1.38). For analyses with TTI ≤ 90 days and TTI ≤ 180 days as outcomes, both expansion and non-expansion states experienced decreases following Medicaid expansion. However, the adjusted difference was not significant for either outcome ([TTI ≤ 90 days adjusted DID: 0.78%; 95% CI: −0.30 to 1.86]; [TTI ≤ 90 days adjusted DID: 0.01%, 95% CI: −0.51 to 0.53]).

Separate analyses by racial group revealed that Non-Hispanic Black, Hispanic, Asian American, and Native Hawaiian or Pacific Islander patients had no relative difference than White patients for all TTI outcomes (≤30 days, ≤90 days, and ≤180 days) following Medicaid expansion.

### 3.5. Refusal of Locoregional Treatment

Unadjusted differences and adjusted DID estimates for changes in refusal of locoregional treatment (refusal of radical proctectomy [RP], refusal of radiation therapy [RT], and refusal of both RP and RT) following Medicaid expansion are presented in Table 2. Parallel trends were observed for refusal of locoregional treatment in the pre-expansion period (2011–2013, *p* > 0.01, Figure 2D). For expansion states, the proportion of patients who refused RP, RT, or both RP and RT decreased following Medicaid expansion. For non-expansion states, the proportion of patients who refused RT increased while the proportion of patients who refused RP decreased. Significant differences between expansion and non-expansion states following ACA implementation were only observed in the proportion of patients refusing RT (adjusted DID: −0.71%; 95% CI: −0.95 to −0.47).

In the analysis with race/ethnicity as the exposure variable, the proportion of Black patients who refused RP decreased, relative to White patients, after Medicaid expansion (adjusted DID: −0.28%; 95% CI: −0.54 to −0.02). Hispanic patients experienced similar decreases in refusal of RT following the ACA (adjusted DID: −0.84%; 95% CI: −1.44 to −0.25). Nevertheless, Asian Americans had an increase in refusal of RP (adjusted DID: 2.16%; 95% CI: 1.16 to 3.15), and no relative differences were observed in refusal outcomes among Native Hawaiian or Pacific Islander patients throughout the study period (*p* > 0.01, Table 3).

## 4. Discussion

In our study of over 112,000 patients diagnosed with incident prostate cancer from 2011 to 2016, we found that ACA implementation was associated with significant increases in Medicaid coverage, decreases in uninsured status, and decreases in refusal of RT. Moreover, upon analysis by specific racial/ethnic subgroup, we found that select racial minorities residing in expansion states experienced larger absolute decreases in uninsured status and refusal of RT regimens than White patients following ACA implementation. These findings corroborate prior studies in showing that Medicaid expansion increased insurance coverage [4,8,28,29], which may have influenced patient-level decisions such as electing to receive RT. Moreover, it is plausible that racial and ethnic disparities in select outcomes narrowed, as our analysis showed that Non-Hispanic Black and Hispanic patients experienced greater decreases in uninsured status and refusal of treatment than White patients following Medicaid expansion; Asian Americans also had greater relative increases in insurance coverage following Medicaid expansion. While this analysis was limited to patients with prostate cancer, the finding that ACA implementation has improved health outcomes for marginalized populations is consistent across numerous studies. For example, among Michigan’s Medicaid expansion enrollees, Black and Hispanic patients reported the largest decrease in days with self-reported poor or fair health than any other race/ethnicity following ACA implementation [30]. Similarly, in other national studies, Black patients living in expansion states experienced larger decreases in mortality from end-stage renal disease and maternal complications during childbirth than White patients following ACA implementation [31,32].

Despite these improvements, though, we also observed that there was no significant association between Medicaid expansion with earlier risk group at diagnosis, time to treatment initiation (TTI) within 180 days, and refusal of locoregional (both RT and RP) treatment. Additionally, all racial minorities residing in expansion states experienced no relative change in TTI relative to White patients after Medicaid expansion. In aggregate, our findings support prior studies in suggesting that insurance coverage alone is not enough to overcome barriers associated with treatment utilization; other avenues of lowering financial toxicity and boosting patient education merit further exploration [33,34,35]. Specifically, rates of refusal of LT and delayed TTI may represent the “tip of the iceberg” regarding issues of access to different elements of prostate cancer care. As a result, policies that modify care delivery processes, in addition to insurance coverage and payment, are necessary to improve population outcomes. These might include implementing community-based interventions to improve access to cancer screenings, increasing minority representation in clinical trials, and more expansive payment incentives that incorporate telemedicine services [36,37,38]. Ultimately, racial disparities in cancer outcomes remain a critical area for research and intervention, and a multifaceted approach to targeting these disparities is needed. For instance, recent reports of Medicaid expansion show that expanded insurance coverage can improve treatment and survival outcomes among low-income and uninsured patients with cancer [39], and that disparities in mortality from gastrointestinal (GI) cancers are reduced among patients residing in expansion states [40]. Specifically, Medicaid expansion was linked to a greater reduction in the 2-year mortality rates among Black individuals with GI cancers, whereas racial disparities in mortality either remained unchanged or worsened in non-expansion states. As more research emerges to evaluate changes in cancer outcomes following Medicaid expansion across a variety of sociodemographic groups, we should ensure that the policies we advocate for are moving the needle towards high-quality cancer care for all.

### Limitations

There are several limitations of this study. First, given that this is an observational study, the DID analysis is limited in its ability to prove any causation. Because randomized experiments of new policies are impractical to implement, we used this DID analysis to minimize as many confounding effects as possible. Second, the NCDB lacks specific patient-level information, including an individual’s eligibility for Medicaid; as a result, we were unable to control for state-level fixed effects. Third, the NCDB is a hospital-based registry and not a population-based registry. The database, however, has significant representation of both community and academic sites across urban and rural localities. Additionally, we note that the 19 states examined in our analysis are from diverse regions. It is likely that state-level comparisons that explore qualitatively the underlying etiologies of these disparities using a lens that parallels comparative health systems would prove illustrative. Fourth, our analysis was limited to patients with prostate cancer aged 40 to 64, which could limit the generalizability of our data, although relatively few patients with prostate cancer will receive a diagnosis under the age of 40. Lastly, there may be potential misclassification, missing data, and selection bias in the database more generally, as well as limited data availability on particular racial subpopulations.

## 5. Conclusions

The effects of Medicaid expansion on prostate cancer outcomes and disparities are still unclear. While ACA implementation was associated with increased insurance coverage and decreased refusal of RT, there was no significant association with earlier risk group at diagnosis, TTI, or refusal of LT. Similarly, racial minorities in expansion states had larger decreases in uninsured status, intermediate-/high-risk disease at presentation, and the refusal of RT regimens than White patients following ACA implementation but experienced no significant changes in TTI. More research is needed to understand how Medicaid expansion affects cancer outcomes and whether these effects are borne equitably among different populations. Future investigations should incorporate longer-term follow-up data and ongoing exploration of how disparities in cancer outcomes following Medicaid expansion vary across a number of disease sites.

## Figures and Tables

**Table 1:** Baseline cohort characteristics among patients residing in Medicaid expansion and non-expansion states. *p*-values were obtained using Pearson’s chi-squared test for categorical variables and the Kruskal–Wallis test for continuous variables.

Sociodemographic or Clinical Characteristic	Expansion State n (%)	Non-Expansion State n (%)	*p*-Value
Total Cohort No.	50,958	61,476	-
**Median Age, years (IQR)**	59 (55–62)	58 (55–62)	<0.001
**Race/Ethnicity**			<0.001
Non-Hispanic White	37,234 (73.07)	42,264 (68.75)	-
Non-Hispanic Black	8091 (15.88)	13,989 (23.76)	-
Hispanic	2395 (4.70)	2408 (3.92)	-
Asian American	742 (1.46)	269 (0.44)	-
Native Hawaiian or Pacific Islander	99 (0.19)	46 (0.07)	-
Other/Unknown	2397 (4.70)	2500 (4.07)	-
**Insurance Status**			<0.001
Private Insurance	44,667 (87.65)	55,365 (86.81)	-
Medicaid	3999 (7.85)	2252 (3.66)	-
Uninsured	1093 (2.14)	2447 (3.98)	-
Other Government	1199 (2.35)	3412 (5.55)	-
**Zip Code-Wide Percent without High School Education**			<0.001
17.6% or more	7554 (14.82)	13,712 (22.30)	-
10.9% to 17.5%	11,655 (22.87)	16,465 (26.78)	-
6.3% to 10.8%	14,825 (29.09)	16,525 (26.88)	-
Less than 6.3%	16,924 (33.21)	14,774 (24.03)	-
**Zip Code-Wide Median Household Income**			<0.001
Less than $40,227	6999 (13.73)	13,558 (22.05)	-
$40,227–$50,353	8739 (17.15)	14,983 (24.37)	-
$50,354–$63,332	11,472 (22.51)	14,106 (22.95)	-
$63,333+	23,748 (46.60)	18,829 (30.63)	-
**Urbanicity**			<0.001
Metro	44,212 (86.76)	50,242 (81.73)	-
Urban	6127 (12.02)	9697 (15.77)	-
Rural	619 (1.21)	1537 (2.50)	-
**Charlson-Deyo comorbidity score**			<0.001
Zero	43,184 (84.74)	51,283 (83.42)	-
One	6492 (12.74)	8720 (14.18)	-
Two	920 (1.81)	1123 (1.83)	-
Greater Than 3	362 (0.71)	350 (0.57)	-
**Facility Type**			<0.001
Community	19,257 (37.79)	33,241 (54.07)	-
Academic	31,701 (62.21)	28,235 (45.93)	-
**Distance to Treatment Facility**			<0.001
0 to < 10 mi	22,921 (44.98)	23,391 (38.05)	-
10 to < 20 mi	11,387 (22.35)	14,259 (23.19)	-
20 to < 50 mi	10,269 (20.15)	13,236 (21.53)	-
≥50 mi	6381 (12.52)	10,590 (17.23)	-
**AUA/ASTRO Risk Group**			0.047
Low	12,471 (24.47)	15,436 (25.11)	-
Intermediate	26,932 (52.85)	32,177 (52.34)	-
High	11,555 (22.68)	13,863 (22.55)	-
**Year of Diagnosis**			0.001
2011	10,576 (20.75)	13,117 (21.34)	-
2012	8779 (17.23)	10,704 (17.41)	-
2013	8149 (15.99)	9996 (16.26)	-
2014	8153 (16.00)	9533 (15.51)	-
2015	8112 (15.92)	9884 (16.08)	-
2016	7189 (14.11)	8242 (13.41)	-

**Table 2:** Association between residence in a Medicaid expansion state and changes in insurance status, risk group at presentation, time from diagnosis to treatment, and refusal of locoregional treatment among men with localized prostate cancer.

	Expansion States			Non-Expansion States				
Characteristic ^a^	Before ^b^	After	Unadjusted Diff. (95% CI)	Before	After	Unadjusted Diff. (95% CI)	Adjusted DID ^c^ (95% CI)	*p*-Value
**Insurance Status**								
Medicaid (%)	5.58	10.5	4.93 (4.47 to 5.40)	3.32	4.09	0.77 (0.47 to 1.07)	4.12 (3.60 to 4.46)	<0.001
Uninsured (%)	2.77	1.42	−1.35 (−1.60 to −1.10)	4.21	3.70	−0.51 (−0.82 to −0.20)	−0.87 (−1.28 to −0.46)	<0.001
**Risk Group at Diagnosis**								
Low Risk (%)	31.5	16.3	−15.2 (−16.0 to −14.5)	31.6	17.2	−14.4 (−15.1 to −13.7)	−0.62 (−1.60 to 0.36)	0.217
Intermediate/High Risk (%)	68.5	83.7	15.2 (14.5 to 16.0)	68.4	82.8	14.4 (13.7 to 15.1)	0.62 (−0.36 to 1.60)	0.217
**TTI**								
≤30 days (%)	10.0	10.7	0.69 (0.16 to 1.22)	12.6	12.5	−0.16 (−0.69 to 0.36)	0.63 (−0.12 to 1.38)	0.101
≤90 days (%)	67.5	66.2	−1.28 (−2.10 to −0.46)	71.6	69.3	−2.22 (−2.95 to −1.50)	0.78 (−0.30 to 1.86)	0.156
≤180 days (%)	94.4	94	−0.35 (−0.76 to 0.05)	95.4	95.0	−0.39 (−0.73 to −0.05)	0.01 (−0.51 to 0.53)	0.969
**Refusal of Treatment**								
Refused RP and RT (%)	0.04	0.02	−0.02 (−0.05 to 0.02)	0.02	0.02	0.00 (−0.02 to 0.03)	−0.02 (−0.06 to 0.02)	0.329
Refused RP (%)	1.10	0.99	−0.11 (−0.29 to 0.07)	0.61	0.58	−0.03 (−0.15 to 0.10)	−0.12 (−0.33 to 0.09)	0.281
Refused RT (%)	1.55	0.96	−0.60 (−0.80 to −0.40)	0.83	0.92	0.09 (−0.06 to 0.24)	−0.71 (−0.95 to −0.47)	<0.001

^a^ Abbreviations: Difference (Diff); 95% Confidence Interval (95% CI); Difference-in-Difference (DID); Time-to-Treatment Initiation (TTI); Radical Prostatectomy (RP); Radiation Therapy (RT). ^b^ Both primary and secondary analyses included patients diagnosed with low-, intermediate-, or high-risk prostate cancer from 1 January 2011 to 31 December 2016. The pre-expansion period was defined as 1 January 2011 to 31 December 2013, and the post-expansion period was defined as 1 January 2014 to 31 December 2016. ^c^ The main independent variables were residence in a Medicaid expansion state, diagnosis in the post-expansion period, and the interaction between the two. All models were adjusted for year of diagnosis, insurance status (where relevant), age, Charlson–Deyo comorbidity coefficient score (CDCC), zip-code wide median household income, zip-code wide education level, facility type (academic research program vs. community cancer program), distance from treatment facility, and rurality (urban vs. rural location).

**Table 3:** Difference-in-difference analysis of changes in insurance status, time from diagnosis to treatment, and refusal of locoregional treatment according to race and ethnicity after Medicaid expansion.

Characteristic ^a^	Before ^b^	Unadjusted % Diff. from White Patients (95% CI)	After	Unadjusted % Diff. from White Patients (95% CI)	Adjusted DID ^c^ (95% CI)	*p*-Value
**Medicaid Insurance Status**						
Non-Hispanic Black (%)	9.67	7.31 (6.92 to 7.71)	14.2	10.1 (9.56 to 10.6)	2.72 (2.08 to 3.35)	<0.001
Hispanic (%)	14.1	11.8 (11.1 to 12.5)	16.5	12.4 (11.5 to 13.3)	0.56 (−0.53 to 1.66)	0.314
Asian American (%)	7.48	5.12 (3.87 to 6.38)	11.1	6.98 (5.05 to 8.90)	2.01 (−0.19 to 4.21)	0.074
NHPI (%)	10.6	8.23 (4.99 to 11.5)	8.33	4.20 (−0.85 to 9.24)	−4.70 (−10.5 to 1.15)	0.116
Non-Hispanic White (%)	2.36	0.00	4.13	0.00	1 [Reference]	N/A
**Uninsured Status**						
Non-Hispanic Black (%)	7.25	5.07 (4.71 to 5.44)	5.19	3.62 (3.29 to 3.95)	−1.47 (−1.96 to −0.98)	<0.001
Hispanic (%)	11.8	9.60 (8.96 to 10.2)	7.59	6.03 (5.45 to 6.60)	−3.47 (−4.34 to −2.59)	<0.001
Asian American (%)	2.60	3.27 (2.07 to 4.47)	2.17	1.04 (−0.16 to 2.23)	−2.12 (−3.85 to −0.39)	0.016
NHPI (%)	2.35	1.91 (−2.92 to 3.28)	3.33	1.77 (−1.37 to 4.91)	1.28 (−3.32 to 5.87)	0.587
Non-Hispanic White (%)	2.17	0.00	1.56	0.00	1 [Reference]	N/A
**Low Risk**						
Non-Hispanic Black (%)	26.8	−6.01 (−6.97 to −5.05)	13.7	−4.13 (−4.93 to −3.32)	2.37 (1.13 to 3.62)	<0.001
Hispanic (%)	30.1	−2.75 (−4.64 to −0.87)	16.6	−1.22 (−2.84 to 0.40)	1.31 (−1.17 to 3.78)	0.301
Asian American (%)	27.6	−5.29 (−9.10 to −1.47)	14.9	−2.91 (−0.66 to 0.76)	2.83 (−2.50 to 8.16)	0.298
NHPI (%)	23.5	−9.31 (−19.3 to 0.68)	8.33	−9.47 (−19.2 to 0.22)	0.57 (−13.7 to 14.9)	0.938
Non-Hispanic White (%)	32.8	0.00	17.8	0.00	1 [Reference]	N/A
**Intermediate/High Risk**						
Non-Hispanic Black (%)	73.2	6.01 (5.05 to 6.97)	86.3	4.13 (3.32 to 4.93)	−2.37 (−3.62 to −1.13)	<0.001
Hispanic (%)	69.9	2.75 (0.87 to 4.64)	83.4	1.22 (−0.40 to 2.84)	−1.31 (−3.78 to 1.17)	0.301
Asian American (%)	72.4	5.29 (1.47 to 9.11)	85.1	2.91 (−0.76 to 6.57)	−2.93 (−8.16 to 2.50)	0.298
NHPI (%)	76.5	9.31 (−0.68 to 19.3)	91.7	9.47 (−0.22 to 19.2)	−0.57 (−14.9 to 13.7)	0.938
Non-Hispanic White (%)	67.2	0.00	82.2	0.00	1 [Reference]	N/A
**TTI ≤ 30 days**						
Non-Hispanic Black (%)	12.1	0.97 (0.32 to 1.63)	12.4	1.03 (0.34 to 1.73)	0.01 (−0.94 to 0.95)	0.991
Hispanic (%)	13.9	2.75 (1.48 to 4.02)	12.0	0.61 (−0.74 to 1.96)	−1.98 (−3.82 to −1.44)	0.035
Asian American (%)	10.2	−0.92 (−3.48 to 1.64)	10.9	−0.53 (−3.58 to 2.52)	0.06 (−3.88 to 4.00)	0.975
NHPI (%)	8.24	−2.89 (−9.58 to 3.80)	18.3	6.92 (−1.13 to 15.0)	7.21 (−3.37 to 17.8)	0.182
Non-Hispanic White (%)	11.1	0.00	11.4	0.00	1 [Reference]	N/A
**TTI ≤ 90 days**						
Non-Hispanic Black (%)	63.3	−8.07 (−9.02 to −7.13)	62.4	−7.30 (−8.30 to −6.29)	0.99 (−0.38 to 2.35)	0.157
Hispanic (%)	65.3	−6.15 (−7.96 to −4.33)	66.2	−3.45 (−5.40 to −1.50)	2.55 (−0.09 to 5.19)	0.058
Asian American (%)	70.2	−1.19 (−4.86 to 2.49)	67.1	−2.54 (−6.95 to 1.87)	−2.27 (−7.94 to 3.40)	0.432
NHPI (%)	69.4	−2.01 (−11.6 to 7.60)	65	−4.68 (−16.3 to 6.96)	−2.76 (−18.0 to 12.5)	0.722
Non-Hispanic White (%)	71.4	0.00	69.7	0.00	1 [Reference]	N/A
**TTI ≤ 180 days**						
Non-Hispanic Black (%)	92.8	−2.81 (−3.26 to −2.37)	92.4	−2.97 (−3.45 to −2.49)	−0.13 (−0.79 to 0.53)	0.701
Hispanic (%)	93.5	−2.05 (−2.88 to −1.22)	93.6	−1.69 (−2.60 to −0.79)	0.19 (−1.04 to 1.41)	0.764
Asian American (%)	93.4	−2.22 (−3.90 to −5.47)	93.1	−2.19 (−4.22 to −0.16)	−0.14 (−2.75 to 2.48)	0.918
NHPI (%)	94.1	−1.47 (−5.84 to 2.90)	93.3	−2.00 (−7.34 to 3.34)	−0.49 (−7.49 to 6.51)	0.890
Non-Hispanic White (%)	95.6	0.00	95.3	0.00	1 [Reference]	N/A
**Refused RP**						
Non-Hispanic Black (%)	1.50	0.86 (0.67 to 0.10)	1.21	0.59 (0.41 to 0.78)	−0.28 (−0.54 to −0.02)	0.038
Hispanic (%)	1.31	0.67 (0.34 to 1.00)	1.10	0.49 (0.15 to 0.83)	−0.13 (−0.61 to 0.34)	0.581
Asian American (%)	0.00	−0.64 (−1.29 to 0.00)	2.13	1.52 (0.76 to 2.28)	2.16 (1.16 to 3.15)	<0.001
NHPI (%)	2.35	1.71 (0.00 to 3.42)	1.72	1.11 (−0.90 to 3.12)	−0.84 (−3.54 to 1.86)	0.542
Non-Hispanic White (%)	0.64	0.00	0.61	0.00	1 [Reference]	N/A
**Refused RT**						
Non-Hispanic Black (%)	1.12	0.07 (−0.15 to 0.28)	1.03	0.16 (−0.04 to 0.37)	0.09 (−0.21 to 0.39)	0.556
Hispanic (%)	2.34	1.29 (0.86 to 1.71)	1.21	0.35 (−0.05 to 0.75)	−0.84 (−1.44 to −0.25)	0.005
Asian American (%)	3.12	2.06 (1.21 to 2.91)	1.68	0.82 (−0.08 to 1.71)	−1.25 (−2.50 to 0.01)	0.051
NHPI (%)	1.19	1.34 (−2.05 to 2.32)	3.39	2.53 (1.60 to 4.89)	2.22 (−1.11 to 5.56)	0.192
Non-Hispanic White (%)	1.06	0.00	0.86	0.00	1 [Reference]	N/A
**Refused Both RP and RT**						
Non-Hispanic Black (%)	0.04	0.02 (−0.01 to 0.05)	0.05	0.03 (0.00 to 0.07)	0.02 (−0.03 to 0.06)	0.465
Hispanic (%)	0.16	0.14 (0.08 to 0.21)	0.00	−0.01 (−0.06 to 0.04)	−0.15 (−0.23 to −0.06)	0.001
Asian American (%)	0.00	−0.02 (−0.13 to 0.09)	0.00	−0.01 (−0.13 to 0.10)	0.01 (−0.16 to 0.17)	0.936
NHPI (%)	0.00	−0.02 (−0.31 to 0.27)	0.00	−0.01 (−0.33 to 0.30)	0.00 (−0.44 to 0.44)	0.989
Non-Hispanic White (%)	0.02	0.00	0.01	0.00	1 [Reference]	N/A

^a^ Abbreviations: Difference (Diff); 95% Confidence Interval (95% CI); Difference-in-Difference (DID); Time-to-Treatment Initiation (TTI); Radical Prostatectomy (RP); Radiation Therapy (RT). Native Hawaiian and Pacific Islander (NHPI). ^b^ Both primary and secondary analyses included patients diagnosed with low-, intermediate-, or high-risk prostate cancer from 1 January 2011 to 31 December 2016. The pre-expansion period was defined as 1 January 2011 to 31 December 2013, and the post-expansion period was defined as 1 January 2014 to 31 December 2016. ^c^ The main independent variables were race/ethnicity, diagnosis in the post-expansion period, and the interaction between the two. All models were adjusted for year of diagnosis, insurance status, age, Charlson–Deyo comorbidity coefficient score (CDCC), zip-code-wide median household income, zip-code-wide education level, facility type (academic research program vs. community cancer program), distance from treatment facility, and rurality (urban vs. rural location).

## Data Availability

Research data from the National Cancer Database are available upon request from the American Cancer Society and the American College of Surgeons (https://ncdbapp.facs.org/puf/).

## Supplementary Materials

The following supporting information can be downloaded at: https://www.mdpi.com/article/10.3390/cancers17030547/s1, Table S1: Sensitivity Analyses of Insurance Status, Risk Group, and Timely Treatment, After Including Early and Late Adopters of Medicaid Expansion.
