# Supplementary material for: Association Between Medicaid Expansion and Insurance Status, Risk Group, Receipt, and Refusal of Treatment Among Men with Prostate Cancer
Source: Cancers (Basel). 2025 Feb 6;17(3):547. doi: 10.3390/cancers17030547 (PMC11817437; doi:10.3390/cancers17030547)
Supplement: Supplementary file 1 [file cancers-17-00547-s001.zip › cancers-3182075-supplementary.pdf]

Table S1. Sensitivity Analyses of Insurance Status, Risk Group, and Timely Treatment, After Including Early and Late Adopters of Medicaid Expansion.

	Expansion States			Non-Expansion States				
**Characteristic**[a]	**Before**[b]	**After**	**Unadjusted Diff. (95% CI)**	**Before**	**After**	**Unadjusted Diff. (95% CI)**	**Adjusted DID**[c] **(95% CI)**	***P* Value**
**Insurance Status**								
Medicaid (%)	4.82	8.96	4.14 (3.83 to 4.45)	3.32	4.09	0.77 (0.47 to 1.07)	3.33 (2.89 to 3.78)	<0.001
Uninsured (%)	2.57	1.36	–1.21 (–1.39 to –1.04)	4.21	3.70	–0.51 (–0.82 to –0.20)	–0.72 (–1.04 to –0.38)	<0.001
**Risk Group at Diagnosis**								
Low Risk (%)	31.9	15.9	–16.0 (–16.6 to –15.5)	31.6	17.2	–14.4 (–15.1 to –13.7)	–1.32 (–2.16 to –0.48)	0.002
Intermediate/High Risk **(%)**	68.1	84.1	16.0 (15.5 to 16.6)	68.4	82.8	14.4 (13.7 to 15.1)	1.32 (0.48 to 2.16)	0.002
**TTI**								
≤30 days (%)	10.2	11.1	0.87 (0.49 to 1.26)	12.6	12.5	–0.16 (–0.69 to 0.36)	0.78 (0.14 to 1.42)	0.016
≤90 days (%)	67.0	66.3	–0.62 (–1.20 to –0.03)	71.6	69.3	–2.22 (–2.95 to –1.50)	0.13 (0.40 to 2.26)	0.005
≤180 days (%)	94.3	94.2	–0.16 (–0.45 to 0.13)	95.4	95.0	–0.39 (–0.73 to –0.05)	0.02 (–0.27 to 0.64)	0.80
**Refusal of Treatment**								
Refused RP and RT (%)	0.03	0.02	0.00 (–0.03 to 0.02)	0.02	0.02	0.00 (–0.02 to 0.03)	–0.01 (–0.04 to 0.02)	0.560
Refused RP (%)	0.96	0.84	–0.11 (–0.23 to 0.01)	0.61	0.58	–0.03 (–0.15 to 0.10)	–0.09 (–2.78 to 0.08)	0.276
Refused RT (%)	1.33	1.15	–0.18 (–0.32 to –0.04)	0.83	0.92	0.09 (–0.06 to 0.24)	–0.29 (–0.50 to –0.07)	0.009
